# Novel saprobic *Hermatomyces* species (Hermatomycetaceae, Pleosporales) from China (Yunnan Province) and Thailand

**DOI:** 10.3897/mycokeys.82.67973

**Published:** 2021-08-09

**Authors:** Guang-Cong Ren, Dhanushka N. Wanasinghe, Jutamart Monkai, Peter E. Mortimer, Kevin D. Hyde, Jian-Chu Xu, Aimin Pang, Heng Gui

**Affiliations:** 1 Center of Excellence in Fungal Research, Mae Fah Luang University, Chiang Rai 57100, Thailand; 2 School of Science, Mae Fah Luang University, Chiang Rai 57100, Thailand; 3 Guiyang Nursing Vocational College, Guiyang 550081, Guizhou, China; 4 Center for Mountain futures, Kunming Institute of Botany, Chinese Academy of Sciences, Honghe County 654400, Yunnan, China; 5 CIFOR-ICRAF China Program, World Agroforestry (ICRAF), Kunming 650201, Yunnan, China; 6 Science and Technology on Aerospace Chemical Power Laboratory, Hubei Institute of Aerospace Chemotechnology, Xiangyang, 441003, Hubei, China

**Keywords:** 2 new species, hyphomycetes, phylogeny, taxonomy, woody litter fungi

## Abstract

During our survey of the diversity of woody litter fungi in China and Thailand, three *Hermatomyces* species were collected from dead woody twigs of *Dipterocarpus* sp. (Dipterocarpaceae) and *Ehretiaacuminata* (Boraginaceae). Both morphology and multigene analyses revealed two taxa as new species (*Hermatomycesturbinatus* and *H.jinghaensis*) and the remaining collections as new records of *H.sphaericus*. *Hermatomycesturbinatus* is characterized by 1) dimorphic conidia, having circular to oval lenticular conidia and 2) turbinate conidia consisting of two columns with two septa composed of 2–3 cells in each column. *Hermatomycesjinghaensis* is characterized by dimorphic conidia, having circular to oval lenticular conidia and clavate or subcylindrical to cylindrical conidia and consisting of one or two columns with 6–8 cells in each column. Phylogenetic analyses of combined LSU, ITS, *tub*2, *tef*1-α and *rpb*2 sequence data supports the placement of these new taxa within Hermatomycetaceae with high statistical support.

## Introduction

Over the past few decades, the number of studies using a molecular-based approach to study microfungal diversity in the greater Mekong subregion (GMS) has increased rapidly, especially on freshwater and woody litter fungi from China (Yunnan Province) and Thailand (Hapuarachchi et al. 2019; Dong et al. 2020; Li et al. 2020; Monkai et al. 2020; Wanasinghe et al. 2020, 2021; Mortimer et al. 2021). Hyde et al. (2018) reported that about 96% of fungi from Thailand are new to science. Feng and Yang (2018) estimated 104,000 fungal species currently exist in Yunnan Province, China; however, only about 6,000 are extant. Therefore, further studies need to be conducted to fill gaps in knowledge regarding the diversity, taxonomy and phylogeny of microfungi in the GMS. Supporting this obligation, we have begun to study plant-based ascomycetes in GMS. The current study accounts for hermatomyces-like ascomycetes recovered from the woody litter in China (Yunnan Province) and Thailand.

*Hermatomyces* was introduced by Spegazzini (1911) with *H.tucumanensis* as the type species. Doilom et al. (2017) accommodated *Hermatomyces* in Lophiotremataceae based on combined LSU, SSU, *tef*1-α and *rpb*2 sequence data. Later, Hashimoto et al. (2017) validated Hermatomycetaceae (Hermatomycetaceae Locq. 1984 was not validly published, Art. 39.1) to accommodate the genus *Hermatomyces*. This genus is known only by its asexual morph that is characterized by sporodochial conidiomata and dimorphic (lenticular or cylindrical) conidia of one or two types. The lenticular conidia are globose to subglobose, hyaline to pale brown peripheral cells with dark brown central cells, and the cylindrical conidia is hyaline, cylindrical to subcylindrical or turbinate and consisting of 1–4 columns of 2–12 cells (Spegazzini 1911; Tibpromma et al. 2016; Hashimoto et al. 2017; Hyde et al. 2019; Pem et al. 2019; Phukhamsakda et al. 2020).

Based on morphological comparisons and phylogenetic affinities, Koukol et al. (2018) revised *Hermatomyces* species and described five new species (viz. *H.bifurcatus*, *H.constrictus*, *H.megasporus*, *H.sphaericoides* and *H.verrucosus*) and one new combination, *H.reticulatus*, from Panama. Accordingly, *H.chromolaenae*, *H.saikhuensis*, *H.tectonae* were treated as *H.sphaericus* and *H.subiculosus*, *H.chiangmaiensis*, *H.thailandicus* were synonymized with *H.reticulatus*, *H.krabiensis* and *H.indicus*, respectively (Koukol et al. 2018). These are probably species complexes that need more detailed study. Subsequent studies introduced *H.bauhiniae*, *H.biconisporus*, *H.clematidis*, *H.trangensis* and *H.truncates* into *Hermatomyces* (Tibpromma et al. 2018; Hyde et al. 2019; Koukol et al. 2019; Nuankaew et al. 2019; Phukhamsakda et al. 2020). Currently, 24 species are recognized in *Hermatomyces* (Koukol et al. 2018, 2019; Nuankaew et al. 2019; Delgado et al. 2020; Phukhamsakda et al. 2020; Table 2).

Our investigation led to the discovery of three *Hermatomyces* species, including two novel species, on dead woody-based substrates. Morphological illustrations and multi-gene phylogenetic analyses with ML, MP and BI of combined LSU, ITS, *tub*2, *tef*1-α and *rpb*2 sequence data are used to confirm the phylogenetic placement of the novel species within *Hermatomyces*.

## Materials and methods

### Sample collection, examination and isolation

Woody litter samples were collected from China (Yunnan Province) during the dry season (December 2019) and Thailand (Tak Province) during the wet season (August 2019). Samples were brought to the laboratory using plastic Ziploc bags. Fungal specimens were then examined using a stereomicroscope (Olympus SZ61, China). Pure cultures were obtained via single spore isolation on potato dextrose agar (PDA) following the methods described in Senanayake et al. (2020). Cultures were incubated at 25 °C for three weeks. Micro-morphological structures were photographed using a Nikon compound microscope (Nikon ECLIPSE Ni) fitted with a Canon (EOS 600D) digital camera. Measurements were taken using the Tarosoft (R) Image Frame Work program. Figures were processed using Adobe Photoshop CS6. Type specimens were deposited in the herbarium of Cryptogams Kunming Institute of Botany Academia Sinica (**KUN-HKAS**). Ex-type living cultures were deposited at the Culture Collection of Mae Fah Luang University (**MFLUCC**) and Kunming Institute of Botany Culture Collection (**KUMCC**).

### DNA extraction, amplification and sequencing

DNA extraction, amplification, sequencing, sequence alignment and phylogenetic analyses follow the methods of Dissanayake et al. (2020) with the following details. Two partial rDNA genes and three protein coding genes were used in our study, including internal transcribed spacer region (ITS) using primer pair ITS5/ITS4 (White et al. 1990), 28S large subunit nuclear ribosomal (LSU) using primer pair LR0R/LR5 (Vilgalys and Hester 1990), translation elongation factor 1-alpha gene (*tef*1-α) using primer pair EF1-983F/EF1-2218R (Rehner and Buckley 2005), RNA polymerase II second largest subunit (*rpb*2) using primer pair fRPB2-5F/fRPB2-7cR (Liu et al. 1999) and β-tubulin (*tub*2) using primer pair T1/T22 (O’Donnell and Cigelnik 1997). Amplification reactions were performed in a total volume of 25 μL of PCR mixtures containing 8.5 μL ddH_2_O, 12.5 μL 2× PCR MasterMix (TIANGEN Co., China), 2 μL DNA template and 1 μL of each primer. The PCR thermal cycle program for LSU, ITS, *tef*1-α and *rpb*2 were set as described in Tibpromma et al. (2018). The PCR amplification condition of *tub*2 was set as denaturation at 94 °C for 3 minutes, followed by 35 cycles of denaturation at 94 °C for 45 seconds, annealing at 56 °C for 50 seconds and extension at 72 °C for 1 minute, with a final extension step at 72 °C for 10 minutes. PCR products were sent to the Qingke Company, Kunming City, Yunnan Province, China, for sequencing. Sequences were deposited in GenBank (Table 1).

**Table 1.** GenBank accession numbers of sequences used for the phylogenetic analyses.

Organism	Strain number	GenBank accession numbers					Reference
		LSU	ITS	*tub*2	*tef*1-α	*rpb*2	
* Anteaglonium globosum *	ANM 925.2^T^	GQ221879	NA	NA	GQ221925	NA	Mugambi and Huhndorf (2009)
* A. parvulum *	MFLUCC 14-0821	KU922915	NA	NA	KU922921	NA	Jayasiri et al. (2016)
* Hermatomyces amphisporus *	CBS 146610	LR812664	LR812664	NA	NA	NA	Delgado et al. (2020)
* H. amphisporus *	CBS 146611	NA	LR812663	LR812674	LR812658	LR812669	Delgado et al. (2020)
* H. amphisporus *	CBS 146612	NA	LR812665	LR812675	LR812659	LR812670	Delgado et al. (2020)
* H. amphisporus *	CBS 146613	LR812662	LR812662	LR812673	LR812657	LR812668	Delgado et al. (2020)
* H. amphisporus *	CBS 146614	LR812666	LR812666	LR812676	LR812660	LR812671	Delgado et al. (2020)
* H. amphisporus *	CBS 146615	LR812667	LR812667	LR812677	LR812661	LR812672	Delgado et al. (2020)
* H. bauhiniae *	MFLUCC 16-0395^T^	MK443378	MK443382	NA	MK443384	MK443386	Hyde et al. (2019)
* H. biconisporus *	KUMCC 17-0183^T^	MH260296	MH275063	NA	MH412771	MH412755	Tibpromma et al. (2018)
* H. bifurcatus *	CCF 5899	LS398262	LS398262	LS398441	LS398416	LS398343	Koukol et al. (2018)
* H. bifurcatus *	CCF 5900^T^	LS398263	LS398263	LS398442	LS398417	LS398344	Koukol et al. (2018)
* H. clematidis *	MFLUCC 17-2085^T^	MT214556	MT310603	NA	MT394735	MT394684	Phukhamsakda et al. (2020)
* H. constrictus *	CCF 5904^T^	LS398264	LS398264	LS398443	LS398418	LS398345	Koukol et al. (2018)
* H. indicus *	MFLUCC 14-1143^T1^	KU764692	KU144920	NA	KU872754	KU712488	Doilom et al. (2017)
* H. indicus *	MFLUCC 14-1144	KU764693	KU144921	NA	KU872755	KU712489	Doilom et al. (2017)
* H. indicus *	MFLUCC 14-1145	KU764694	KU144922	NA	KU872756	KU712490	Doilom et al. (2017)
* H. iriomotensis *	KH 361^T^	LC194367	LC194483	NA	LC194394	LC194449	Hashimoto et al. (2017)
*** H. jinghaensis ***	**HKAS 112167^T^**	**MW989519**	**MW989495**	**NA**	**MZ042642**	**NA**	**This study**
* H. krabiensis *	MFLUCC 16-0249^T^	KX525742	KX525750	NA	KX525758	KX525754	Tibpromma et al. (2016)
*H.krabiensis* (*H.chiangmaiensis*)	MFLUCC 16-2817 ^T2^	KY559394	NA	NA	NA	NA	Tibpromma et al. (2017)
* H. megasporus *	CCF 5897	NA	LS398265	LS398444	LS398419	LS398346	Koukol et al. (2018)
* H. megasporus *	CCF 5898^T^	LS398266	LS398266	LS398445	LS398420	NA	Koukol et al. (2018)
* H. nabanheensis *	KUMCC 16-0149^T^	KY766059	KY766058	NA	KY766061	NA	Hyde et al. (2017)
* H. pandanicola *	MFLUCC 16-0251^T^	KX525743	KX525751	NA	KX525759	KX525755	Tibpromma et al. (2016)
* H. reticulatus *	CCF 5893	LS398267	LS398267	LS398446	LS398421	LS398347	Koukol et al. (2018)
*H.reticulatus* (*H.subiculosus*)	MFLUCC 15-0843^T3^	KX259523	KX259521	NA	KX259527	KX259529	Hyde et al. (2016)
* H. sphaericoides *	CCF 5896	NA	LS398271	LS398448	LS398425	LS398351	Koukol et al. (2018)
* H. sphaericoides *	CCF 5908^T^	LS398273	LS398273	LS398450	LS398427	LS398352	Koukol et al. (2018)
* H. sphaericoides *	CCF 5907	NA	LS398272	LS398449	LS398426	NA	Koukol et al. (2018)
* H. sphaericoides *	CCF 5895	LS398270	LS398270	LS398447	LS398424	LS398350	Koukol et al. (2018)
* H. sphaericus *	PMA 116080	LS398281	LS398281	LS398454	LS398431	LS398356	Koukol et al. (2018)
* H. sphaericus *	PMA 116081	NA	LS398283	LS398455	LS398432	LS398357	Koukol et al. (2018)
* H. sphaericus *	PRM 946201	NA	LS398284	LS398456	LS398433	LS398358	Koukol et al. (2018)
* H. sphaericus *	PRC 4116	NA	LS398275	NA	NA	NA	Koukol et al. (2018)
* H. sphaericus *	PRC 4105	NA	LS398286	NA	NA	NA	Koukol et al. (2018)
* H. sphaericus *	PRC 4104	NA	LS398278	LS398453	LS398430	LS398355	Koukol et al. (2018)
* H. sphaericus *	PRC 4100	NA	LS398277	LS398452	LS398429	LS398354	Koukol et al. (2018)
* H. sphaericus *	PRC 4106	NA	LS398279	NA	NA	NA	Koukol et al. (2018)
* H. sphaericus *	PMA 116085	NA	LS398280	NA	NA	NA	Koukol et al. (2018)
* H. sphaericus *	PMA 116082	NA	LS398285	NA	NA	NA	Koukol et al. (2018)
* H. sphaericus *	KZP 462	NA	LS398287	LS398457	LS398434	LS398359	Koukol et al. (2018)
* H. sphaericus *	PRC 4117	NA	LS398276	NA	NA	NA	Koukol et al. (2018)
*H.sphaericus* (*H.chromolaenae*)	MFLUCC 16-2818^T4^	KY559393	NA	NA	NA	NA	Tibpromma et al. (2017)
*H.sphaericus* (*H.saikhuensis*)	MFLUCC 16-0266^T5^	KX525740	KX525748	NA	KX525756	KX525752	Tibpromma et al. (2016)
*H.sphaericus* (*H.saikhuensis*)	MFLUCC 16-0267	KX525741	KX525749	NA	KX525757	KX525753	Tibpromma et al. (2016)
*H.sphaericus* (*H.tectonae*)	MFLUCC 14-1140^T6^	KU764695	KU144917	NA	KU872757	KU712486	Doilom et al. (2017)
*H.sphaericus* (*H.tectonae*)	MFLUCC 14-1141	KU764696	KU144918	NA	KU872758	NA	Doilom et al. (2017)
*H.sphaericus* (*H.tectonae*)	MFLUCC 14-1142	KU764697	KU144919	NA	NA	KU712487	Doilom et al. (2017)
*** H. sphaericus ***	**MFLUCC 21-0036**	**MW989516**	**MW989492**	**MZ042643**	**MZ042639**	**MZ042636**	**This study**
*** H. sphaericus ***	**KUMCC 20-0231**	**MW989517**	**MW989493**	**MZ042644**	**MZ042640**	**MZ042637**	**This study**
* H. trangensis *	BCC 80741^T^	KY790600	KY790598	NA	KY790606	KY790604	Nuankaew et al. (2019)
* H. trangensis *	BCC 80742	KY790601	KY790599	NA	KY790607	KY790605	Nuankaew et al. (2019)
* H. tucumanensis *	CCF 5912	LS398288	LS398288	LS398458	LS398435	LS398360	Koukol et al. (2018)
* H. tucumanensis *	CCF 5913	LS398289	LS398289	LS398459	LS398436	LS398361	Koukol et al. (2018)
* H. tucumanensis *	CCF 5915	LS398290	LS398290	LS398460	LS398437	LS398362	Koukol et al. (2018)
*** H. turbinatus ***	**MFLUCC 21-0038^T^**	**MW989518**	**MW989494**	**MZ042645**	**MZ042641**	**MZ042638**	**This study**
* H. verrucosus *	CCF 5903^T^	LS398292	LS398292	LS398462	LS398439	LS398364	Koukol et al. (2018)
* H. verrucosus *	CCF 5892	LS398291	LS398291	LS398461	LS398438	LS398363	Koukol et al. (2018)

### Sequence alignment and phylogenetic analyses

Representative species used in the phylogenetic analyses were selected based on previous publications (Koukol et al. 2018; Nuankaew et al. 2019; Delgado et al. 2020; Phukhamsakda et al. 2020). Sequences were downloaded from GenBank (http://www.ncbi.nlm.nih.gov/), and their accession numbers are listed in Table 1. The newly generated sequences in this study were assembled by BioEdit 7.0.9.0 (Hall 1999). Individual gene regions were separately aligned in MAFFT v.7 web server (http://mafft.cbrc.jp/alignment/server/) (Katoh et al. 2019). The alignments of each gene were improved by manually deleting the ambiguous regions plus gaps and combined using BioEdit 7.2.3. Final alignments containing LSU, ITS, *tub*2, *tef*1-α and *rpb*2 were converted to NEXUS format (.nxs) using CLUSTAL X (2.0) (Thompson et al. 1997) and processed for Bayesian and maximum parsimony analysis. The FASTA format was changed into PHYLIP format via the Alignment Transformation Environment (ALTER) online program (http://www.sing-group.org/ALTER/) and used for maximum likelihood analysis (ML).

ML was carried out in CIPRES Science Gateway v.3.3 (http://www.phylo.org/portal2/; Miller et al. 2010) using RAxML-HPC2 on XSEDE (8.2.12) (Stamatakis 2014) with the GTRGAMMA substitution model and 1,000 bootstrap iterations. Maximum parsimony analysis (MP) was performed in PAUP v. 4.0b10 (Swofford 2002) with the heuristic search option and Tree-Bisection-Reconnection (TBR) of branch-swapping algorithm for 1,000 random replicates. Branches with a minimum branch length of zero were collapsed, and gaps were treated as missing data (Hillis and Bull 1993).

Bayesian analysis was executed in MrBayes v.3.2.2 (Ronquist et al. 2012). The model of evolution was estimated using MrModeltest v. 2.3 (Nylander et al. 2008) via PAUP v. 4.0b10 (Ronquist and Huelsenbeck 2003). The SYM+I+G for LSU and ITS; HKY+I for *tub*2; GTR+I+G for *tef*1-α and *rpb*2 were used in the final command. Markov chain Monte Carlo sampling (MCMC) in MrBayes v.3.2.2 (Ronquist et al. 2012) was used to determine posterior probabilities (PP) (Rannala and Yang 1996; Zhaxybayeva and Gogarten 2002). Bayesian analyses of six simultaneous Markov chains were run for 2,000,000 generations and trees were sampled and printed to output at every 200 generations (resulting in 10,001 total trees). The first 25% of sampled trees were discarded as part of the burn-in procedure, the remaining 7,501 trees were used to create the consensus tree and the average standard deviation of split frequencies was set as 0.01.

Phylogenetic trees were visualized in FigTree v1.4.0 (http://tree.bio.ed.ac.uk/software/figtree/; Rambaut 2012), the tree was edited using Microsoft PowerPoint before being saved in PDF format and finally converted to JPG format using Adobe Illustrator CS6 (Adobe Systems, USA). The finalized alignments and trees were deposited in TreeBASE, submission ID: TB2:S28514 (http://purl.org/phylo/treebase/phylows/study/TB2:S28514).

Ex-type strains are indicated with superscript “T”, and newly generated sequence is shown in bold. NA represents sequences that are unavailable in GenBank. Abbreviations:

**ANM** A.N. Miller;

**BCC**BIOTEC Culture Collection, Bangkok, Thailand;

**CBS**Centraal Bureau voor Schimmel cultures, Utrecht, The Netherlands;

**CCF** Culture Collection of Fungi, Charles University, Prague, Czech Republic;

**HKAS** The herbarium of Cryptogams Kunming Institute of Botany Academia Sinica;

**KH** K. Hirayama;

**KUMCC** Culture Collection of Kunming Institute of Botany, Kunming, China;

**KZP** O. Koukol;

**MFLUCC** Mae Fah Luang University Culture Collection, Chiang Rai, Thailand;

**PMA**Herbarium of the University of Panama, Panama City, Panama;

**PRC**Herbarium of the Charles University, Prague, Czech Republic;

**PRM** Herbarium of the National Museum, Prague, Czech Republic.

**T1** Type of *Hermatomycesthailandicus*;

**T2** Type of *H.chiangmaiensis*;

**T3** Type of *H.subiculosus*;

**T4** Type of *H.chromolaenae*;

**T5** Type of *H.saikhuensis*;

**T6** Type of *H.tectonae*.

## Results

### Phylogenetic analysis

The phylogenetic analysis was conducted using 57 strains in Hermatomycetaceae, and two outgroup taxa *Anteagloniumglobosum* (ANM 925.2) and *A.parvulum* (MFLUCC 14-0821) in Pleosporales (Table 1). The aligned sequence matrix comprised five gene regions (LSU: 887 bp, ITS: 530 bp, *tub*2: 606 bp, *tef*1-α: 952 bp and *rpb*2: 1,028 bp) and a total of 4,003 characters (including gaps), of which 3,207 characters were constant, 174 variable characters were parsimony-uninformative and 622 characters were parsimony-informative. The Kishino-Hasegawa test shows length = 1,388 steps with CI = 0.671, RI = 0.884, RC = 0.593 and HI = 0.329. The RAxML analysis of the combined dataset yielded a best scoring tree with a final ML optimization likelihood value of -13406.555506. Estimated base frequencies were as follows: A = 0.241874, C = 0.266701, G = 0.257552, T = 0.233873; substitution rates AC = 1.188604, AG = 4.826453, AT = 1.273226, CG = 0.855218, CT = 11.409386, GT = 1.00; gamma distribution shape parameter α = 0.16102.

**Figure 1.**
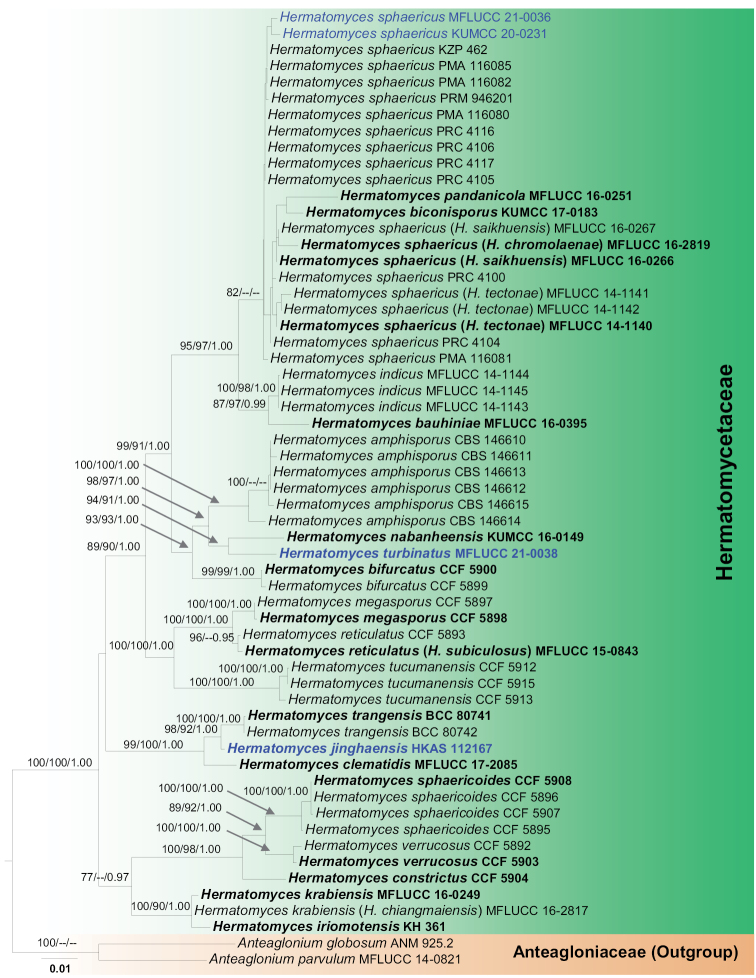
Phylogenetic RAxML tree based on analysis of a combined LSU, ITS, *tub*2, *tef*1-α and *rpb*2 and dataset. Bootstrap support values for ML and MP equal to or higher than 75% and Bayesian PP equal to or greater than 0.95 are shown at nodes. Hyphens (--) represent support values less than 75% / 0.95 BYPP. The ex-type strains are in bold and the new isolate in this study is in blue bold. The tree is rooted with *Anteagloniumglobosum* (ANM 925.2) and *A.parvulum* (MFLUCC 14-0821). The scale bar represents the expected number of nucleotide substitutions per site.

In the phylogenetic tree obtained from ML, MP and BI analysis (Fig. 1) the maximum likelihood analysis resulted in trees largely with similar topology and clades as in the maximum parsimony and Bayesian analyses. The new species, *Hermatomycesturbinatus*, is sister to *H.nabanheensis* (KUMCC 16-0149) with high support (94% ML, 91% MP and 1.00 BYPP, Fig. 1). *Hermatomycesjinghaensis* is nested between *H.trangensis* and *H.clematidis* with a strongly supported monophyletic group (98% ML, 92% MP, 1.00 PP; Fig. 1). New isolates of *H.sphaericus* (KUMCC 20-0231; MFLUCC 21-0036) clustered with remaining *H.sphaericus* strains as a monophyletic group (Fig. 1). The topology of the phylogenetic tree is in accordance with recent phylogenetic studies discussing species in Hermatomycetaceae (Nuankaew et al. 2019; Phukhamsakda et al. 2020).

### Taxonomy

#### Hermatomyces turbinatus

Taxon classificationFungiPleosporalesHermatomycetaceae

G.C. Ren & K.D. Hyde sp. nov.

479CDBD8-11CB-58C0-93ED-BED46E6A7908

558166

Facesoffungi Number No: FoF09735

Figure 2

##### Etymology.

Referring to the turbinate shape of the conidia.

##### Holotype.

HKAS 112724.

##### Description.

*Saprobic* on woody litter of *Dipterocarpus* sp. (Dipterocarpaceae) **Sexual morph** Undetermined. **Asexual morph***Colonies* on natural substrate forming sporodochial conidiomata, superficial, scattered, small groups, circular or oval, sterile mycelial outer zone enclosing a black-brown velvety margin, sparse, black sporulating center, shiny, glistening, circular or oval, conidia readily liberated when agitated. *Mycelium* superficial, branched, septate, hyaline to pale brown, 2–3 μm wide. *Conidiophores* 6–8 × 2–3 μm, micronematous, straight or flexuous, smooth, short, pale brown. *Conidiogenous cells* 3–5 × 2–3 μm, monoblastic, integrated, terminal, determinate, often arising directly on the superficial mycelium, subsphaerical, ovoid or ampulliform, hyaline to pale brown, smooth finely verruculose. *Conidia* dimorphic, solitary, smooth-walled. *Lenticular conidia* 24–30 × 17–21 μm (x = 27 × 20 μm, n = 20), 12–15 μm thick, thick-walled, circular to oval in front view, smooth, solitary, muriform, central cells dark brown to black, peripheral cells hyaline to pale brown, forming a weakly ring, sometimes slightly constricted at septa, obovoid or oblong in lateral view, arranged in 2 rows, a row of composed of 4–6 cells, end cells pale brown to hyaline, middle cells dark brown. *Turbinate conidia* turbinate, pyriform, 27–36 μm in length, 19–28 µm wide in broadest part of lower cells, (x = 32 × 23 μm, n = 20), asymmetrical with the upper cells smaller than lower cells, thick-walled, smooth, septate, constricted distinct at septa, consisting of two columns with two septa composed of 2–3 rectangular to globose cells in each column, usually upper part of terminal cells dark brown, becoming hyaline towards the lower side, two cells hyaline in the lower cells swollen with oil globules.

**Figure 2.**
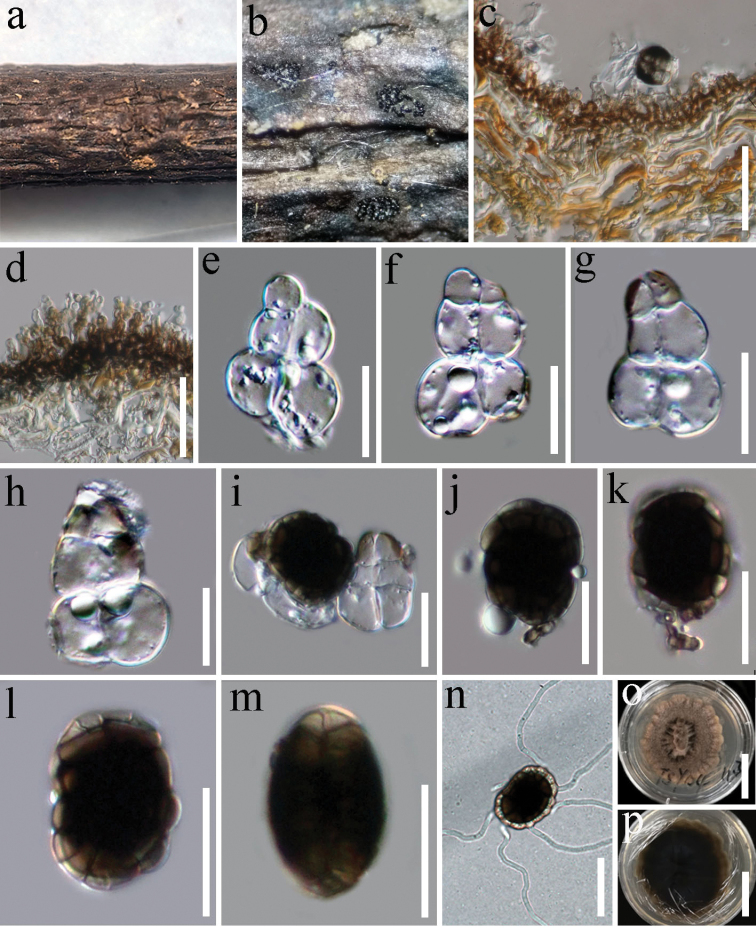
*Hermatomycesturbinatus* (HKAS 112724, holotype) **a, b** sporodochia on natural substrate **c** vertical section of sporodochium **d** conidiophores and conidiogenous cells **e–h** turbinate conidia **i** turbinate and mature lenticular conidia **j–m** mature lenticular conidia **n** germinated conidium **o, p** culture characters on PDA. Scale bars: 30 μm (**c**); 20 μm (**d–n**); 30 mm (**o, p**).

##### Known host and distribution.

*Dipterocarpus* sp. (Thailand).

##### Culture characteristics.

*Colonies on PDA*, reaching 30–40 mm diam., after 3 weeks at 25–30 °C, circular, convex with papillate and radially furrowed at the center, rough, labate, crenate edge, fluffy, dense, gray black, in reverse darkens at the center, pale brown to gray at edge.

##### Material examined.

Thailand, Tak Province. Ban Na Sam Ngao District, on woody litter of *Dipterocarpus* sp. (Dipterocarpaceae), 22 August 2019, G. C. Ren, TSY04 (HKAS 112724, ***holotype***), ex-type living culture, MFLUCC 21-0038.

##### Notes.

*Hermatomycesturbinatus* is introduced as a new species based on its distinct morphology, which is supported by phylogenetic analyses. In the phylogenetic analyses, *H.turbinatus* is distinct from extant species in this genus and formed a sister clade to *H.nabanheensis* with strong support (94% ML, 91% MP, 1.00 PP; Fig. 1). *Hermatomycesturbinatus* differs from *H.nabanheensis* in having turbinate conidia with two columns, while *H.nabanheensis* has cylindrical conidia with one or two columns. *Hermatomycesturbinatus* has two conidial types, and its lenticular conidia are similar to *H.tectonae* in shape and size. However, the turbinate conidia of *H.turbinatus* have 2 columns of 2–3 cells in each column, while the turbinate conidia of *H.tectonae* have 2 columns of 3 cells in each column. We also compared the morphological characters of *H.turbinatus* to other species of *Hermatomyces* (Table 2). Despite no molecular data being available for the three species viz. *H.dimorphus*, *H.uniseriatus* and *H.truncates*, *H.turbinatus* nonetheless differs from these species in conidial characteristics (Table 2).

**Table 2.** Synopsis of the morphological characteristics of *Hermatomyces* species.

Species	Lenticular conidia size (μm)	Cylindrical / turbinate conidia feature			Host	Country	Reference
		Shape	Length × width (μm)	Number of columns (cells)			
* Hermatomyces amphisporus *	27–36(–38) × 18–29(–31)	Cylindrical, pyriform or turbinate	30‒38 × 20‒26	2(–4) (6–12 cells)	*Cyathea* sp., *Sabalminor*	Mexico, USA	Castañeda-Ruiz and Heredia (2000); Delgado et al. (2020)
* H. bauhiniae *	25–36 × 15–20	Cylindrical	20–28 × 8–11	1 (2–3-septate)	* Bauhinia variegata *	Thailand	Hyde et al. (2019)
* H. biconisporus *	28–34 × 15–25	Cylindrical	32–39 × 14.5–26	1–2 (3–4 cells)	*Pandanus* sp.	China	Tibpromma et al. (2018)
* H. bifurcatus *	(24–)30–36.5(–41) × (18–)21.5–26(–28)	Cylindrical	Apex: 7–16 × 7–12 Basal: 9–14 × 13–18.5	2 (2–3 cells)	Unknown	Panama	Koukol et al. (2018)
* H. chromolaenae *	9.2–10.4 × 10.2–11.5	NA	NA	NA	* Chromolaena odorata *	Thailand	Tibpromma et al. (2017)
* H. clematidis *	30–45 × 24–31	Cylindrical	29–35 × 12–14	1–2 (5–6 cells)	* Clematis sikkimensis *	Thailand	Phukhamsakda et al. (2020)
* H. constrictus *	(22–)25.5–29.5(–32) × 19–23.5(–27.5)	Cylindrical	Lower cells: (20–)24–30.5(–37) × 12–17 Upper cells: (16–)20–26(–30) × 8–14	1 (2 cells)	* Bauhinia cumanensis *	Panama	Koukol et al. (2018)
* H. dimorphus *	35‒55 × 15‒20	Cylindrical	15‒40 × 10‒15	4 (7 cells)	Unknown	India	Rao and de Hoog (1986)
* H. indicus *	18‒30 × 11.5‒19	Turbinate	22.4‒35.4 × 11.4‒21.6	2 (6–7 cells)	* Phoenix rupicola *	India	Prasher and Prasher (2014)
* H. iriomotensis *	30–36 × 20–27	Cylindrical	20.5–33 × 7–12.5	1–2 (3–7 cells)	Unknown	Japan	Hashimoto et al. (2017)
*** H. jinghaensis ***	**30–40 × 25–30**	**Clavate, subcylindrical**	**33–43 × 11–13**	**1–2 (6–8 cells)**	**Unknown**	**China**	**This study**
* H. krabiensis *	24.3–32.5 × 12.1–21.3	Cylindrical	20.4–26.4 × 8.6–19.7	1–2 (2–3 cells)	* Pandanus odorifer *	Thailand	Tibpromma et al. (2016)
* H. megasporus *	(45–)49–56(–59) × (31–)37–46	Cylindrical	(37–)49.5–60.5(67–) × 18–28(–32)	2 ((5–)6–7(–10) cells)	Unknown	Panama	Koukol et al. (2018)
* H. nabanheensis *	20.2–25.1 × 16.6–20.7	Cylindrical	15.3–26.8 × 12.1–18.2	1–2 (2–3 cells)	*Pandanus* sp.	China	Hyde et al. (2017)
* H. pandanicola *	12–15.7 × 20–30.1	Cylindrical	13.2–20.6 × 8.9–11.9	2 (2 cells)	* Pandanus odorifer *	Thailand	Tibpromma et al. (2016)
* H. reticulatus *	3–40(–45) × 25–34(–41)	NA	NA	NA	Unknown	Thailand, Panama	Hyde et al. (2016); Koukol et al. (2018)
* H. saikhuensis *	14.2–21.4 × 11.2–19.3	NA	NA	NA	* Pandanus odorifer *	Thailand	Tibpromma et al. (2016)
* H. sphaericoides *	(20.5–)24.5–28(–31) × (20–)23–26(–29)	NA	NA	NA	Unknown	Panama	Koukol et al. (2018)
*H.sphaericus* (PMA 116080)	(21–)24–29(–32.5) × (18–)21–27(–31.5)	NA	NA	NA	Various host plants	Tropical or subtropical	Koukol et al. (2018)
*** H. sphaericus ***	**27–29 × 26–28**	NA	NA	NA	***Dipterocarpus* sp., *Ehretiaacuminata***	**China, Thailand**	**This study**
* H. tectonae *	(23–)26–29(–33) × (19–)23–25(–28)	Cylindrical	(27–)31–32(–35) ×(21–)23	2 (6 cells)	* Tectona grandis *	Thailand	Doilom et al. (2017)
* H. trangensis *	27.5‒35 × 25‒32.5	NA	NA	NA	* Arenga pinnata *	Thailand	Nuankaew et al. (2019)
* H. truncates *	(26–)31.5–36.5(–37) × 22–27(–30)	Cylindrical	Lower cells: 14–22.5(–28) × 8.5–14.5	1 (2–3 cells)	* Averrhoa carambola *	Ghana, Panama	Koukol et al. (2019)
			Upper cells: 12–18(–30) × (6–)8–12.5				
* H. tucumanensis *	(22–)27–35 × 18–25	Obclavate or subcylindrical	(21–)23–26(–28.5) × 7–14	2 (3–6 cells)	Unknown	Panama	Koukol et al. (2018)
*** H. turbinatus ***	**24–30 × 17–21**	**Turbinate**	**27–36 × 19–28**	**2 (2–3 cells)**	***Dipterocarpus* sp.**	**Thailand**	**This study**
* H. uniseriatus *	27–36 × 15.5–24	Cylindrical	19–34 × 10–12.5	1 (3–4 cells)	* Smilax campestris *	Argentina	Leão-Ferreira et al. (2013)
* H. verrucosus *	23–30(–39) × 21–29.5	NA	NA	NA	Unknown	Panama	Koukol et al. (2018)

NA: absent

#### Hermatomyces jinghaensis

Taxon classificationFungiPleosporalesHermatomycetaceae

G.C. Ren & K.D. Hyde sp. nov.

586F538B-54BB-5D95-A10A-50FE2A43AEE0

558165

Facesoffungi Number No: FoF09736

Figure 3

##### Etymology.

The species epithet “*jinghaensis*” refers to the location where the species was collected.

##### Holotype.

HKAS 112167.

##### Description.

*Saprobic* on unidentified woody litter. **Sexual morph** Undetermined. **Asexual morph***Colonies* on natural substrate forming sporodochial conidiomata, superficial, scattered, small groups, circular, sterile mycelial outer zone enclosing a black velvety margin, dense, thick, black sporulating center, shiny, glistening, circular or oval, conidia readily liberated when agitated. *Mycelium* superficial, branched, septate, hyaline to pale brown, 2–3 μm wide. *Conidiophores* 30–45 × 2–3 μm, mononematous, cylindrical, straight or flexuous, smooth, pale brown. *Conidiogenous cells* 4–6 × 2–3 μm, monoblastic, integrated, terminal, determinate, often arising directly on the superficial mycelium, cylindrical, ampulliform, hyaline to pale brown, smooth finely verruculose. *Conidia* dimorphic solitary, smooth-walled. *Lenticular conidia* 30–40 × 25–30 μm (x = 37 × 28 μm, n = 20), 21–25 μm thick, thick-walled, circular to oval in front view, smooth, solitary, muriform, central cells brown to dark brown, peripheral cells hyaline to subhyaline, forming a wide and distinct ring, sometimes slightly constricted at septa, obovoid or oblong in lateral view, central cells brown to dark brown, peripheral cells pale brown to brown. *Cylindrical conidia* 33–43 μm in length, 11–13 µm wide in broadest part of lower cells (x = 39 × 12 μm, n = 20), clavate or subcylindrical, straight or flexuous, septate, constricted distinct at the septa, with large guttules, consisting of one or two columns, each column with 6–8 cells, apical cell rectangular to globose, smooth, hyaline, smooth, basal cells acute, rectangular to cylindrical, pale brown.

**Figure 3.**
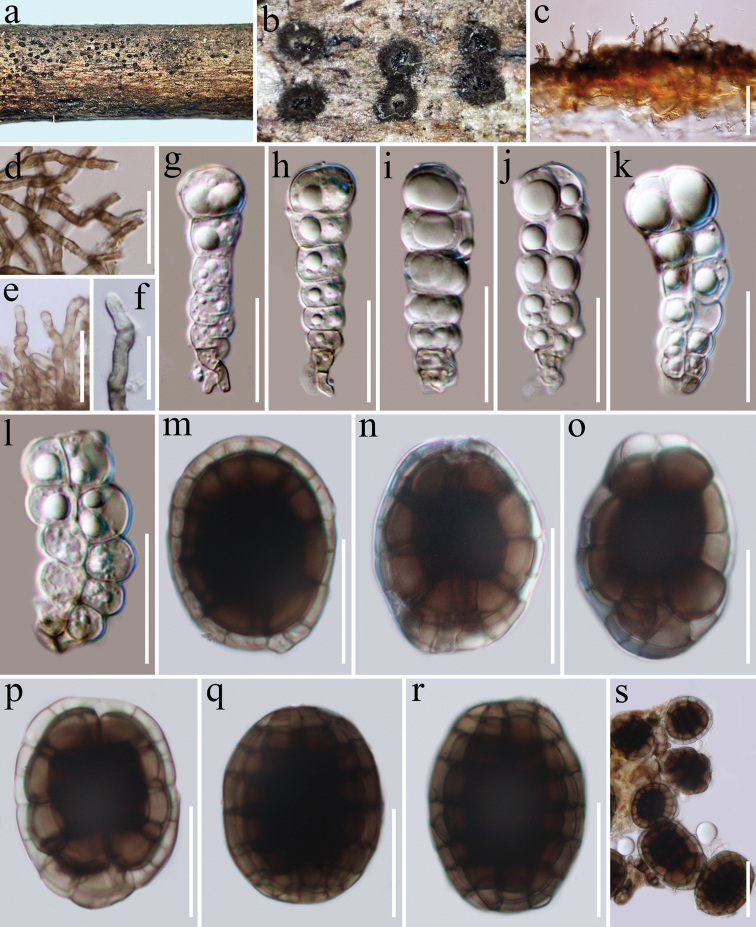
*Hermatomycesjinghaensis* (HKAS 112167, holotype) **a, b** sporodochia on natural substrate **c** vertical section of sporodochium **d** conidiophores **e, f** conidiogenous cells **g–l** cylindrical conidia **m–s** mature lenticular conidia. Scale bars: 50 μm (**c**); 30 μm (**d**); 20 μm (**e–r**); 30 μm (**s**).

##### Known host and distribution.

Unidentified woody litter (China)

##### Material examined.

China, Yunnan Province, Xishuangbanna Dai Autonomous Prefecture, Jinghong, Jingha (21°78.06'N, 101°05.61'E), on unidentified woody litter, 19 December 2019, D.N. Wanasinghe, DW57 (HKAS 112167, ***holotype***), no living culture.

##### Notes.

*Hermatomycesjinghaensis* is introduced as a new species based on its distinct morphology and the phylogenetic results of a combined LSU, ITS, *tub*2, *tef*1-α and *rpb*2 dataset. *Hermatomycesjinghaensis* nested with *H.clematidis* and *H.trangensis* in a strongly supported monophyletic group (99% ML, 100% MP, 1.00 PP; Fig. 1). *Hermatomycesjinghaensis* is characterized by both lenticular and cylindrical conidia. *Hermatomycesjinghaensis* differs from *H.clematidis* in having cylindrical conidia with one or two columns, each of which has 6–8 cells with large guttules, while the latter has 5–6 cells for each column conidia. *Hermatomycestrangensis* differs from *H.jinghaensis* in having only lenticular conidia.

#### Hermatomyces sphaericus

Taxon classificationFungiPleosporalesHermatomycetaceae

(Sacc.) S. Hughes 1953.

272AC737-BC12-5F42-AA42-C0262ADA5FD2

298410

Facesoffungi Number No: FoF05259

Figure 4

##### Description.

*Saprobic* on woody litter of *Dipterocarpus* sp. (Dipterocarpaceae) and *Ehretiaacuminata* (Boraginaceae). **Sexual morph** Undetermined. **Asexual morph***Colonies* on natural substrate forming sporodochial conidiomata, superficial, circular or irregular, scattered or crowded, consisting of a velvety, dense, annular, gray brown, sterile mycelial outer zone and a black, glistening, abundantly sporulating granulose center, with conidia readily liberated when agitated. *Mycelium* 2–2.5 μm wide, superficial, composed of a tightly network of branched, septate, smooth or finely verruculose, hyaline or pale brown hyphae. *Conidiophores* 10–13 × 2–4 μm (x = 12 × 3 μm, n = 10) micronematous, cylindrical or forked, smooth, hyaline or pale brown, often corresponding to conidiogenous cells. *Conidiogenous cells* 5–8 × 3–5 μm (x = 7 × 4 μm, n = 20), monoblastic, integrated, terminal, cylindrical, hyaline to pale brown, smooth or finely verruculose. *Conidia* of one type, 27–29 × 26–28 μm (x = 28 × 27 μm, n = 30) μm, 19–24 μm thick, solitary, lenticular, globose, subglobose in front view, muriform, smooth, central cells brown, dark brown, outer ring of peripheral cells narrow, pale brown to brown, often constricted at septa, disk-shaped in lateral view, consisting of two rows, each row with 4–6 cells, hyaline to light brown at lower and upper cells, middle cells brown to black brown.

**Figure 4.**
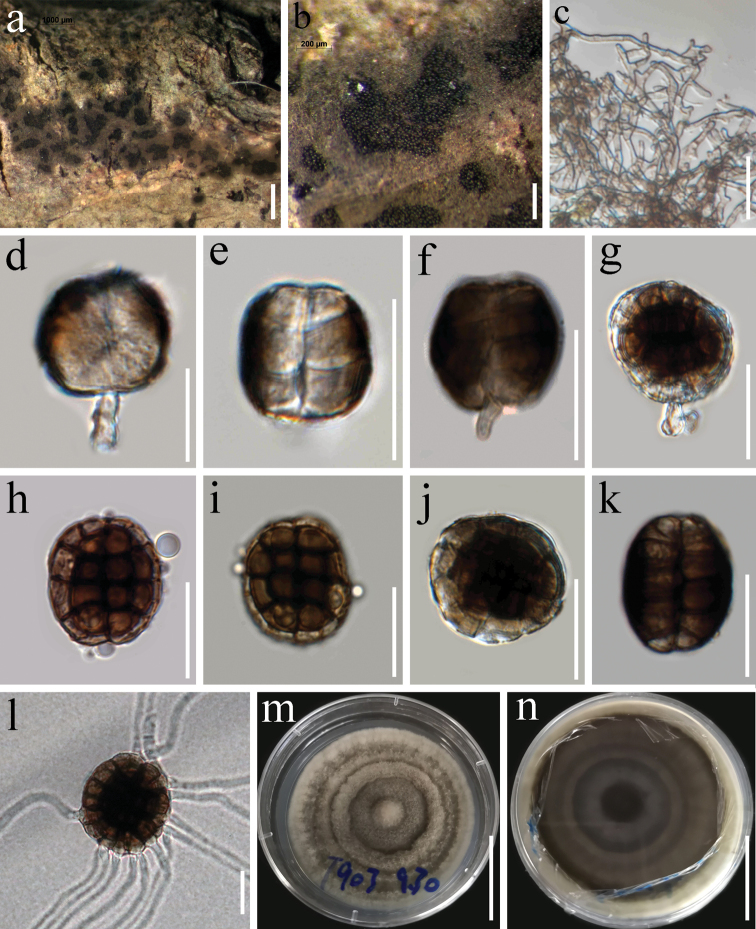
*Hermatomycessphaericus* (HKAS 112725) **a, b** colonies on the natural substrate **c** mycelia **d–g** young conidia **h–k** mature conidia (**h–j** surface view **k** thickness view) **l** germinated conidium **m, n** culture characters on PDA. Scale bars: 1000 μm (**a**); 200 μm (**b**); 20 μm (**c–i, l**); 30 μm (**j, k**); 3 cm (**m, n**).

##### Known host and distribution.

Tropical and subtropical regions of Central and South America, Africa, Asia, Oceania and North America. The species were found as saprobes on Acanthaceae, Apocynaceae, Arecaceae, Asteraceae, Dipterocarpaceae, Euphorbiaceae, Fabaceae, Lamiaceae, Leguminosae, Mimosaceae, Nyctaginaceae, Oxalidaceae, Pandanaceae, Pinaceae, Rhamnaceae, and Sterculiaceae (Zhang et al 2009; Koukol et al. 2018, 2019).

##### Culture characteristics.

Colonies on PDA, reaching 35–40 mm diam., after 3 weeks at 25–30 °C, with circular, umbonate, fluffy, velvety, entire edge, a circular raised band, gray white, in reverse dark gray, black toward the center.

##### Material examined.

Thailand, Tak Province, Tha Song Yang District, on woody litter of *Dipterocarpus* sp. (Dipterocarpaceae), 22 August 2019, G. C. Ren, T903 (HKAS 112725), living culture, MFLUCC 21-0036; China, Yunnan Province, Xishuangbanna (21°55.19'N, 101°15.24'E), on woody litter of *Ehretiaacuminata* (Boraginaceae), 4 August 2020, G. C. Ren, JH39 (HKAS 112166), living culture, KUMCC 20-0231.

##### Notes.

The characters of our new strain of *Hermatomycessphaericus* (KUMCC 20-0231, MFLUCC 21-0036) are similar to the type collection (K(M)–IMI 37763) in having gray black to black sporodochia, mononematous, pale brown, smooth, monoblastic, integrated, terminal, cylindrical, hyaline to pale brown conidiogenous cells and globose to subglobose conidia (Hughes 1953). A multigene phylogeny indicates that novel strains clustered within the *H.sphaericus* clade (Fig. 1). We name our strain (KUMCC 20-0231, MFLUCC 21-0036) as *H.sphaericus*, which has been reported from different plant families and genera (Koukol et al. 2018). However, we consider this might be a species complex that need further detailed studies. Our study provides the new host records of *H.sphaericus* on *Dipterocarpus* sp. (Dipterocarpaceae) and *Ehretiaacuminata* (Boraginaceae), and updates sequence data for the new collections of *H.sphaericus*.

## Discussion

This study introduces two new species of woody-based litter fungi; *Hermatomycesjinghaensis* from Yunnan, China and *Hermatomycesturbinatus* on *Dipterocarpus* sp. from Thailand. We also report for the first time two new records of *H.sphaericus* on *Dipterocarpus* sp. and *Ehretiaacuminata* in China and Thailand.

*Hermatomyces* (Hermatomycetaceae) is different from other similar genera in its sporodochial conidiomata and in having one to two (lenticular and cylindrical conidia) unusual conidial types (Spegazzini 1911). All species of *Hermatomyces* have lenticular conidia with similar characteristics, whereas some species have cylindrical and turbinate conidia, which have greater variance in shape, size, number of columns and cells. Koukol et al. (2018, 2019) have reported that multiple species may occur together on a single sample, a phenomenon we observed, which may complicate morphological identification and separation for culturing. Therefore, molecular sequence data are more reliable for the identification of *Hermatomyces* species (Tibpromma et al. 2016, 2017, 2018; Nuankaew et al. 2019; Phukhamsakda et al. 2020).

*Hermatomycessphaericus* was introduced by Hughes (1953), which may be the most widespread of species in *Hermatomyces* distributed across many subtropical and tropical regions worldwide (Wijayawardene et al. 2014; Doilom et al. 2017; Koukol et al. 2018, 2019; Hyde et al. 2019; Jayasiri et al. 2019; Nuankaew et al. 2019; Phukhamsakda et al. 2020). This species has been reported as saprobic on dead plant tissues of several host families (Tibpromma et al. 2016, 2017; Doilom et al. 2017; Jayasiri et al. 2019). In addition, Koukol et al. (2018) reported that *H.sphaericus* (ARIZ: PS0053) was isolated from seeds of *Apeibamembranacea* (Malvaceae), suggesting this species could be an endophyte. Previous studies have indicated that *H.sphaericus* is not restricted to any single host (Koukol et al. 2018, 2019; Jayasiri et al. 2019), whereas other species of *Hermatomyces* are saprobic on a limited number of hosts and are limited to specific regions (Rao and de Hoog 1986; Leão-Ferreira et al. 2013; Prasher and Prasher 2014; Hyde et al. 2016, 2017, 2019; Tibpromma et al. 2016, 2017, 2018; Doilom et al. 2017; Hashimoto et al. 2017; Koukol et al. 2018, 2019; Nuankaew et al. 2019; Delgado et al. 2020; Phukhamsakda et al. 2020; Table 2). In this study, our new strains of *H.sphaericus* had slight morphological differences in lenticular conidia size compared to the type strains and other strains of *H.sphaericus* (Hughes 1953, Table 2). As reported by Koukol et al. (2018), *H.sphaericus* is a plurivorous species, and accordingly the phenotypic variation among strains could be influenced by environmental factors and culture conditions or it could have speciated in isolated polulations (Hyde et al. 2020).

Species delineation in *Hermatomyces*, especially in the *H.sphaericus* clade, is subject to much controversy due to species inconsistency in morphological and phylogenetic status. Koukol et al. (2018) synonymized *H.chromolaenae*, *H.saikhuensis* and *H.tectonae* under *H.sphaericus* based on morphological and molecular comparisons and suspected that *H.pandanicola* could either be a hybrid species or incorrect sequences were used in the analysis. Koukol et al. (2019) considered that during isolation of *H.biconisporus*, a conidium of *H.sphaericus* might have been taken instead, leading to contamination when extracting DNA and the misinterpretation of its taxonomic placement. Phukhamsakda et al. (2020) further confirmed that *H.biconisporus*, *H.pandanicola* and *H.sphaericus* should be treated as the same species based on Genealogical Concordance Phylogenetic Species Recognition (GCPSR) analysis.

*Hermatomyces* had long been treated as “*incertae sedis*” within Ascomycota (Wijayawardene et al. 2012). Doilom et al. (2017) placed *Hermatomyces* in Lophiotremataceae baed on phylogenetic analyses, and consequently, Hashimoto et al. (2017) revised the family Lophiotremataceae based on morphological observations and phylogenetic analyses, and *Hermatomyces* was accepted in the family Hermatomycetaceae, as monophyletic. Recent studies and our study indicate *Hermatomyces* to be highly polyphyletic, and *Hermatomyces* morphology has evolved, which is mainly characterized by lenticular and cylindrical conidia (Fig. 1; Koukol et al. 2018, 2019; Hyde et al. 2019; Phukhamsakda et al. 2020). Support for a single *H.sphaericus* species (Fig. 1) lacks internal statistical support and includes *H.biconisporus*, *H.chromolaenae*, *H.pandanicola*, *H.saikhuensis* and *H.tectonae* and we suspect that this is a species complex. Tibpromma et al. (2018) also noted that *H.sphaericus* could be a species complex including several species and did not accept the synonymy of *H.saikhuensis* and *H.tectonae* in *H.sphaericus* owing to their significant base-pair differences.

In this study, we combined two non-translated loci (LSU, ITS) and three protein-coding regions (*tub*2, *tef*1-α and *rpb*2) to carry out phylogenetic analysis for *Hermatomyces* species in order to validate phylogenetic placement of the taxa within *Hermatomyces*. In our phylogenetic analyses, *H.tectonae*, *H.chromolaenae*, *H.biconisporus*, *H.pandanicola* and *H.saikhuensis* grouped together with strains of *H.sphaericus* (PRC 4100, PRC 4104, PMA 116081). *Hermatomycessaikhuensis* and *H.chromolaenae* are characterized by one conidium type (lenticular) similar to *H.sphaericus*, however, they differ in the shape, color and size of conidia (Tibpromma et al. 2016, 2017; Table 2). *Hermatomycestectonae*, *H.biconisporus* and *H.pandanicola* are characterized by dimorphic conidia which differ from *H.sphaericus* (Tibpromma et al. 2016, 2018; Doilom et al. 2017; Koukol et al. 2018; Table 2). *Hermatomycessphaericus* (PRC 4100, PRC 4104, PMA 116081) did not have a morphological description for inter-species comparison (Koukol et al. 2018). Further taxon sampling and more sequence data are needed to elucidate this clade.

## Supplementary Material

XML Treatment for Hermatomyces turbinatus

XML Treatment for Hermatomyces jinghaensis

XML Treatment for Hermatomyces sphaericus
